# Between-match variation of peak match running intensities in elite football

**DOI:** 10.5114/biolsport.2022.109456

**Published:** 2021-10-25

**Authors:** Bradley Thoseby, Andrew D. Govus, Anthea C. Clarke, Kane J. Middleton, Ben J. Dascombe

**Affiliations:** 1 Sport and Exercise Science, School of Allied Health, Human Services and Sport, La Trobe University, Australia; 2 High-Performance Department, Melbourne City Football Club, Melbourne, Australia; 3 Applied Sport Science and Exercise Testing Lab, University of Newcastle, Australia

**Keywords:** Team sports, Football, Soccer, Variability, Peak match running intensities, Peak running intensities

## Abstract

Peak match running intensities have recently been introduced to quantify the peak running demands of football competition, across incremental time intervals, to inform training practices. However, their between-match variation is yet to be comprehensively reported, limiting the ability to determine meaningful changes in peak match running intensities. The current study aimed to quantify the between-match variability in peak match running intensities across discrete moving average durations (1–10 min). GPS data were collected from 44 elite football players across 68 matches (mean ± SD; 13 ± 10 observations per player). For inclusion players must have completed 70mins of a match across a minimum of two matches. Performance metrics included total and high-speed (> 19.8 km · h^-1^) running distances and average acceleration (m · s^-2^), expressed relative to time. For each metric, the coefficient of variation and smallest worthwhile difference were calculated. The peak match running intensity data was similar to previously reported data from various football competitions. The between-match CV of relative total distance ranged between 6.8–7.3%, with the CV for average acceleration and relative high-speed running being 5.4–5.8% and 20.6–29.8%, respectively. The greater variability observed for relative high-speed running is likely reflective of the varying constraints and contextual factors that differ between matches. The reported between-match variability helps to provide context when interpreting match performance and prescribing training drills using peak match running intensity data.

## INTRODUCTION

While physical performance is a key outcome measures across all sports, quantifying the variance surrounding selected performance metrics is not common, despite such information helping provide crucial context when interpreting the data [1]. The available data suggest that athletic performance is highly variable in both endurance and power-based sporting competitions [2]. However, little is known about the inherent variability of team sport performance due to the challenges in measuring running performance and quantifying contextual factors associated with match play [3]. Changes in performance (i.e. the “signal”) should be interpreted relative to the total variance (i.e. the “noise”) present in the metric to determine whether the observed change in performance is real or an artefact of biological, statistical or measurement error [1]. Furthermore, quantifying the within and between-variation in physical performance measures allows for the effects of contextual factors (e.g., competition travel, level of opposition and time of season), ergogenic strategies and training programs to be thoroughly investigated [2]. To date, the majority of literature has focused on reporting performance variability within individual sports, typically involving time-trials, fixed distance or weightlifting events [2]. However, such individual sports are closed events which are vastly different to team sports such as football, where variability in physical demands is introduced through both technical and tactical elements as well as external opposition. Consequently, the unpredictable nature of football match play provides inherent between-match variation within performance metrics that reflect the various contextual factors.

Additionally, the overall variation of physical performance is also influenced by both extrinsic and intrinsic factors. Intrinsic factors that contribute to the variation around performance metrics may include circadian rhythms, psychological readiness and arousal levels [4, 5], whereas extrinsic factors may include environmental conditions, quality of opposition, and time between fixtures [6–8]. Further, the usefulness of match running performance metrics can be largely influenced by the accuracy, validity and reliability of the relevant technology employed. For example, GPS technology is the primary technology used in field-based team sports to quantify both training and match running demands [9]. However, GPS technology has its own inherent variation, with the coefficient of variation (CV) being heavily affected by running speed [10]. For example, when completing straight line shuttle runs using various locomotor speed (walk, jog, run, sprint), GPS devices demonstrated acceptable variation for total distance (TD) covered (CV [90% confidence interval (CI): 1.9% [1.6–2.3]) compared to a criterion radar system [11]. However, there was considerable greater variation in the GPS data when the analysis was constrained to only high-speed (> 15 km **·** h^-1^) (CV: 4.7% [4.0–5.8]) and very high-speed running (> 20 km **·** h^-1^) (CV: 10.5% [9.0–12.5]) [11]. Despite these limitations, GPS technology remains a primary tool that is employed to measure the physical performance demands of field-based team sports due to their practicality and ability to collect and record bulk data from multiple sensors [10]. Match analysis of football has typically reported on the TD covered either as an average of the entire match duration or at various running speed thresholds [12]. However, more recent analysis has employed the use discrete moving average durations (e.g., 1–10 min) to assess the peak match running intensities throughout a match in an effort to identify the greatest physical demands placed on field-based team sport athletes [13].

The quantification of the peak match running intensities also offers value in the prescription of training stimuli designed to replicate match day requirements (e.g. small-sided games or football-based conditioning drills). Often external training loads are prescribed with the intention of accumulating volume across a variety of GPS based metrics, i.e., TD, high-speed distance (HSD) and accelerations, however, the training design used to attain these loads may not reflect the intensity of match play. Consequently, the development and prescription of specific training drills relative to the greatest in-match physical demands may be more appropriate [13]. While the data in isolation presents with some contextual limitations, the peak match running intensities across a 1–10 minute moving average duration helps inform the prescription of training practices that reflect the intensity of match play. The use of a moving average duration has shown to be superior to fixed durations when quantifying peak running demands, with fixed durations underestimating peak running demands by ˜7–25% dependent upon metric and epoch duration [14, 15]. Though the usefulness of such analysis to inform specific football conditioning has been questioned [3], such data helps provide ecological validity of prescribing and assessing training intensities against match play. As such providing more contextual clarity and relevance than simply applying the total match or discrete period average demands.

Recent literature has questioned the usefulness of peak match running intensities to inform training, due to the high variability associated with the metrics (TD: 6.2% CV, HSD [> 19.8 km **·** h^-1^]: 25.2% CV, Sprint Distance [> 25.2 km **·** h^-1^]: 46.1% CV) [3]. However, there was no consideration given to the typical variability of total match derived measures of physical output, previously reported to be 2.4–4.3% CV for TD [16, 17], with HSD (> 19.8 km · h^-1^) and sprint distances (> 25.2 km · h^-1^) displaying variations of 16.2–18.1% and 30.8–38.9% CV, respectively [7, 18]. While Novak, Impellizzeri (3) reported that the peak match running intensities possessed slightly greater variability, this analysis was limited to a single 3-min period with no exploration of whether the magnitude of variation was affected by peak match intensity duration.

Anecdotally, it could be suggested that the variation of 1-min peak match running demands would demonstrate greater variability due to the temporal changes in sprinting and high-speed running demands relative to time than a 10-min epoch, which would incorporate more running and periods of recovery. As such, it’s likely that the spectrum of peak match intensity epochs would be affected by temporals shifts in running demands which would be reflected through differing levels of between-match variation. Anecdotally, it could be suggested that the variation of peak match running intensities at a 1 min epoch would differ to a 10 min epoch due to the differing physical demands associated with these durations of play [13]. As such, it is important to consider the broad spectrum of variability across a range of epochs to gain a full insight into the variability of peak match running intensities.

Practically, the development of specific athlete physiological capacities, e.g., anaerobic power or repeat sprint ability occurs through the prescription of small-sided games [19] and, as such, the use of peak match intensities to guide training prescription can be useful for informing drill selection, drill constraints and drill durations. While the available data using peak running intensities has grown rapidly, it is acknowledged there is a current gap in the literature quantifying the influence of contextual factors and technical involvements during peak running periods. In order to interpret longitudinal changes in the peak match running intensities and prescribe training stimuli reflective of match play, it is first necessary to understand the between-match variability associated with these metrics. Accurate quantification of the variation in these measures across durations reflective of training drill durations, i.e., 1–10 min, will allow for a more robust analysis of typical match performance and help determine “meaningful changes” in between-match performance, while also allowing for more specific training load prescription. With peak match running intensities presenting a physical target for players to hit during specific training drills, understanding the variability of the metric allows practitioners to adjust set targets to encompass a larger proportion of “typical” peak match running intensities. Therefore, this study aimed to quantify the between-match variation in the peak match running intensities observed for elite football players for durations ranging between 1 to 10 minutes.

## MATERIALS AND METHODS

### Experimental Overview

The peak match running intensities of elite football players observed across durations of 1–10 min were determined using an observational study design. Durations of 1–10 minutes were selected for analysis as per previously reported [20]. Match data were collected from 44 elite football players across 68 matches that were played across three seasons of the Hyundai A-League (2015–2018). This resulted in a total of 494 individual match observations (mean ± SD; 13 ± 10 observations per player, range; 2–43 observations). To be included in the analyses, players must have played at least two matches where they performed for a minimum of 70 minutes to avoid any data skewing from the impact of substitutions. Past research has demonstrated that substitutes have different peak running demands during match-play to starting players [14], with the largest proportion of substitute introductions occurring after ˜70 minutes [21, 22]. This resulted in a total of 494 individual match observations (mean ± SD; 13 ± 10 observations per player). All participants played for the same team, with data representative of the entire playing group. Goalkeepers were excluded from analysis due to their vastly different match demands. Informed consent and institutional ethics approval were attained prior to the commencement of the study (HREC#: 18056).

### Activity Profile

Players’ match activities were recorded using portable 18 Hz GPS units (STATSports, Belfast, Northern Ireland) that were worn in a custom-made harness underneath the playing jersey located between the scapulae. These GPS devices have previously been determined as valid and accurate in tracking athlete movements, with the bias for distance and velocity measures reported as 1.15–2.02% [23]. Athletes consistently wore their own identical GPS device between matches to avoid any inter-unit variability, with satellite availability > 10 for all analysed matches. Raw GPS data were downloaded post-match using relevant proprietary software (STATSports, Northern Ireland) and then exported into R Studio statistical programming software (RStudio, V 1.1.453). Running speed data points that exceeded 10 m **·** s^-2^ and acceleration speeds above ± 6 m **·** s^-2^ were replaced with zero values, which due to the nature of data analysis outlined below had negligible effects on observed values [13].

From the available data, three metrics of match running performance were selected for analysis of their peak match intensities: relative TD covered (m **·** min^-1^); relative HSD covered (> 19.8 km **·** h^-1^) (m **·** min^-1^) and; average acceleration (AveAcc) (m **·** s^-2^). Average acceleration was calculated through summing the absolute acceleration and deceleration speeds and averaging them over a defined time duration to provide an indication of the total acceleration requirements of match-play [24]. From these metrics, peak match running intensities were quantified using a moving average technique, across ten incremental durations (i.e. 1–10 min), using R Studio statistical programming software (RStudio, V 1.1.453), and custom-made code, with the maximum value obtained from each variable at each time period being recorded.

### Statistical Analysis

Analyses were conducted in R Studio statistical software (V 1.2.1335) using the *lme4* package (V 1.1–21) [25]. Prior to analysis, assessment of data normality and identification of outliers was conducted via the inspection of boxplots and quantile-quantile plots. Data were subset by intensity period (10 levels: 1–10 min), with separate linear mixed models conducted to calculate the coefficient of variation (CV) for each intensity period for each response variable (relative TD, relative HSD, AveAcc), yielding ten models per response variable. Crossed random intercepts for both athlete ID and match date were included to assess the average between-match variability for each athlete. Data were log transformed and then back-transformed and converted to a percentage to express the between-match changes in peak match running intensities as a CV (%) with imprecision presented as a 95% confidence interval (CI). Additionally, the smallest worthwhile difference (SWD) was calculated for each time point to determine the smallest practically meaningful between match difference in peak running intensities. The smallest worthwhile difference was calculated as 0.3 × within-subject variance and then doubled, to account for the small amount of error associated with GPS technology, with data presented in raw units.

## RESULTS

All data were deemed normally distributed by visual inspection of a Quantile-Quantile plot, with no outliers owing to measurement error detected. Data points that were outliers but represented real data (i.e., not due to measurement error) were included in analysis. Peak match running intensities for each performance metric are presented in Table 1 below. The between-match variability of relative TD was low across all discrete epochs (CV: 6.8–7.3%, Table 1), as was the between-match variability of AveAcc across all epochs (CV: 5.4–5.8%, Table 1) The between-match variability in relative HSD was higher across all epochs (CV: 20.6–29.8%, Table 1), with variability gradually increasing with epoch duration.

**TABLE 1:** Variability measures of peak match running intensities across 1–10 min moving average durations.

Performance Metric		Time Period									
		1 min	2 min	3 min	4 min	5 min	6 min	7 min	8 min	9 min	10 min
Relative Distance (m · min^-1^)	Mean ± SD	195 ± 18	165 ± 14	153 ± 13	147 ± 13	142 ± 12	139 ± 12	136 ± 12	134 ± 12	132 ± 12	130 ± 12
	SWD	7.4	5.5	4.7	4.4	4.2	4.1	3.9	3.9	3.9	3.9
	CV %	6.9	6.8	6.8	6.8	6.9	7.0	7.3	7.2	7.2	7.2
	95% CI	5.3–8.9	5.2–8.7	5.3–8.7	5.3–8.7	5.4–8.9	5.5–8.9	5.7–9.3	5.7–9.3	5.7–9.3	5.7–9.3

Relative HSD (m · min^-1^)	Mean ± SD	60 ± 17	36 ± 11	28 ± 9	23 ± 7	21 ± 7	19 ± 6	17 ± 6	16 ± 5	15 ± 5	14 ± 5
	SWD	8.2	4.9	3.8	3.2	2.8	2.5	2.3	2.2	2.0	1.9
	CV %	20.6	22.4	24.6	26.2	26.9	27.3	27.9	28.9	29.8	29.6
	95% CI	15.4–27.4	17.0–29.8	18.6–32.6	19.9–34.8	20.4–35.6	20.8–36.1	21.2–36.9	21.9–38.3	22.7–39.5	22.5–39.3

Average Acceleration (m · S^-2^)	Mean ± SD	1.09 ± 0.09	0.92 ± 0.07	0.85 ± 0.07	0.81 ± 0.06	0.79 ± 0.06	0.77 ± 0.06	0.75 ± 0.06	0.74 ± 0.06	0.73 ± 0.06	0.72 ± 0.06
	SWD	0.042	0.031	0.029	0.027	0.025	0.025	0.023	0.023	0.022	0.022
	CV %	5.4	5.5	5.5	5.5	5.6	5.7	5.6	5.7	5.8	5.7
	95% CI	4.1–7.0	4.3–7.2	4.2–7.2	4.2–7.1	4.3–7.3	4.4–7.3	4.4–7.3	4.4–7.4	4.4–7.5	4.4–7.4

Note: SD; standard deviation, SWD; smallest worthwhile difference, CV; coefficient of variation, 95% CI; 95% confidence intervals for coefficient of variation, HSD; high-speed distance.

## DISCUSSION

The current study quantified the between-match variation in the peak match running intensities of elite football players, across moving average durations of 1–10 min, to allow the effect of contextual factors and ergogenic practices on match running performance to be explored. The primary findings demonstrate that the peak match running intensities of both relative TD and AveAcc were stable across 1–10 minute epochs, whereas the relative HSD demonstrated high variability that increased with epoch length. Importantly, these are the first data to report upon the between-match variability of the acceleration demands of football match play. These findings not only provide critical context for the analysis of immediate and longitudinal peak match running intensity data, but also provide context for the prescription of training loads during match-specific conditioning sessions.

The between-match variability of the relative TD peak match running intensities was demonstrated to be stable across all moving average durations (CV: 6.8–7.3%). This supports past data that has quantified the between-match variability of the absolute TD covered (CV: 2.4–6.1%) across elite football, rugby and Australian football match-play [17, 26, 27]. Importantly, it is also similar to that previously reported for a 3 min window peak match running intensities (CV: 6.2%) [3]. However, research has shown that irrespective of contextual factors, such as environmental conditions, level of opposition or match outcome, that the TD covered across a match is largely unchanged [28, 29]. More specifically, the main differences in physical performance are likely better reflected in fluctuations in HSD, with the between-match variability of absolute HSD being considerably high (CV: ˜16–30%) [7]. In the current study, the between-match variability of the relative HSD peak match running intensities (CV: 20.6–29.6%) was similar to past data for a sole 3 min window (CV: 25.2%) [3]. Further, it was also similar to that previously reported for total HSD and sprint distance in football (CV: 16.2–38.9%) [7, 18]. The greater variability associated with relative HSD reflects the multi-faceted nature of the collective variability (i.e., that which is introduced through measurement, sampling and biological error). Firstly, GPS devices demonstrate high variability at running speeds > 14.4 km **·** h^-1^ when compared to a criterion radar system [11]. Additionally, due to the relatively low proportion of HSD when compared to TD, small changes in the HSD between matches are reflected through larger changes in variability due to the smaller cluster size. Due to this sensitivity, the tactical strategies of the team will also provide a source of variability, with different oppositions and match play situations likely affecting the playing style of the team.

This current study is the first to assess the between-match variability of AveAcc demands in football, with the AveAcc metric stable across all moving average durations (CV: 5.4–5.8%). The amalgamation of acceleration and deceleration activities is a novel method in assessing the propulsive and braking requirements of match-play, both of which place higher energy demands on the athlete [24]. Due to the relative infancy of the metric, the underlying properties associated with the variability of this metric are not yet fully understood. However, a primary factor associated with the AveAcc variability would be the inconsistencies associated with GPS technology in the quantification of acceleration profiles. Despite research showing that the GPS technology possesses good inter-unit reliability for AveAcc (CV: 3.6 ± 1.5%), this was still three times higher than the inter-unit reliability of other GPS devices (CV: 1.2 ± 1.5%) [30]. Despite this, in contrast to the present study, acceleration parameters have been identified as the most variable physical output metric [31], across both halves and entire matches [32–34]. However, these collective research investigations reported on the quantification of acceleration counts, rather than the AveAcc quantified across discrete time points. When quantifying acceleration counts, what constitutes an acceleration or deceleration can be largely affected by whether or not the “raw” or “processed” GPS data is used, as well the calculations implemented by proprietary software to clean the data [30, 35]. Therefore, the use of an AveAcc metric may be more representative of match intensity and allow for better comparisons between data sets as well as data obtained across GPS devices.

Importantly, this study presents the most comprehensive analysis of between-match variation of peak match running intensities in team sports. When comparing between studies, the present data has presented the between-match variability across ten moving average epochs, i.e., 1–10 min, rather than a singular duration (3 min) as reported upon by Novak, Impellizzeri (3). As such, the current is the first to report upon the changes in between-match variability with various window lengths for physical performance metrics. Further, the current study limited its analysis to starting players that completed at least 70 minutes of a match to limit the impact of substitution on maximum physical intensities [14]. It is acknowledged, however, that the data set was collected from a single football team and factors such as tactical formation, athletes’ physical capacities, and opposition tactics were not directly accounted for in the present study. Further to this, a wealth of data has reported that the different playing positions possess significantly different match running demands [6, 34, 36], with additional contextual factors such as time of season, environmental conditions, and time between fixtures shown to alter physical output [8, 37, 38]. As such, it is important for future research to account for these factors when comprehensively measuring the between-match variation in peak intensity match performance metrics.

With practitioners regularly using total match data in the preparation of athletes for competition, peak match running intensities should not be overlooked. While it is acknowledged that the understanding and application of peak match running intensity is evolving, dismissal of the metric as a whole is precarious. While it is argued that using peak match intensities data to inform training only prepares athletes for the average peak match demands, understanding the variability of the metric can provide a more specific target range from which to prescribe drills. For example, the lower limit of the peak match intensity spectrum could be targeted on lighter days, or conditioning sessions may target the higher limit of peak match running intensities (mean ± CV). This may allow for the frequent targeting of peak match running intensities without compromising match-day performance. As such, an understanding of the inherent variation of the reported physical output metrics may allow for the better and replication of match demands during training across a wider range of intensities.

## CONCLUSIONS

The present study provides important information on the variability of the peak match intensities that are emerging as a common tool in assessing physical match performance. The quantification of variance in the analysis of peak match intensities for these measures is imperative in providing context to the data and maximising the ecological validity and practicality of its use. Such context would allow coaches to distinguish between meaningful and non-meaningful changes in peak match running intensities at both an individual and team levels, helping to directly compare between-match physical performance. It has previously been suggested that the variability of peak match running intensities limits their use in informing training intensities [3]. However, the between-match variability reported for peak running intensities in the present study was only slightly higher (TD: ˜2–3% and HSD: ˜2.5–11.5%) to that previously reported for total match demands which have historically been used in prescribing training volumes and intensities. As such, the use of peak match running intensities to inform training practices should not be overlooked.

## Disclosure Statement

No potential conflict of interest was reported by the authors.
